# Editorial: Promoting motor development in children in the COVID-19 era: Science and applications

**DOI:** 10.3389/fpubh.2022.988085

**Published:** 2022-09-22

**Authors:** Nancy Getchell, Patrizia Tortella, Guido Francesco Fumagalli, Arja Sääkslahti

**Affiliations:** ^1^Department of Kinesiology and Applied Physiology, University of Delaware, Newark, DE, United States; ^2^Department of Education, Free University of Bozen-Bolzano, Bolzano, Italy; ^3^Department of Diagnostics and Public Health, Pharmacology Section, University of Verona, Verona, Italy; ^4^Faculty of Sport and Health Sciences, University of Jyväskylä, Jyväskylä, Finland

**Keywords:** motor development, pandemic (COVID-19), physical activity, children, education

At the dawn of 2020, the World Health Organization (WHO) announced the presence of a novel strain of coronavirus; by March, WHO declared that COVID-19 met the criteria of a pandemic (globally widespread and sustained disease transmission among humans). The implications of a global pandemic were immediate and profound. For many children, the COVID-19 pandemic meant that they were confined indoors for an indefinite amount of time. They could no longer go to school in-person, play on their local sports teams, or congregate in parks and on playgrounds. The consequences of the COVID-19 pandemic on motor development have the potential to be profound on infants and children of all ages, and to have a lasting impact on many distinct aspects of development for years to come. It is for that reason that we felt it imperative to share research related to the direct and indirect impacts of COVID-19 on motor development. We chose to explore this topic as broadly as possible. The nine publications included in this Research Topic represent a wide range of subjects, participant ages, as well as represented countries, making it a truly global examination of the impact of COVID-19 on the development of motor skills.

Two studies focus on the differences that emerged during the COVID-19 pandemic in the development of infants (Huang et al.) and young children (Auilar-Farias et al.). In Huang et al., the authors explore associations among motor and communication characteristics in born prior to and during the COVID-19 pandemic, arguing that the social isolation experienced by the latter group may lead to motor and communication delays. In fact, they determined that infants who experienced social isolation during the pandemic had an increased risk of compromised neurodevelopment, but only if the infant was first born. This suggests that siblings played a vital role in maintaining infant motor development, which in turn underscores the importance of interactions with other children to avoid motor delays. The next study by Aguilar-Faris and colleagues, also explores the behavioral and emotional changes that resulted from the pandemic in toddlers and young children in Chile. They surveyed almost 2,000 caregivers about changes in their children's emotional and motor behaviors along with their own levels of stress. They found that children significantly reduced their physical activity while concomitantly increasing screen time; further, children's sleep quality declined in the early pandemic. Further (and not surprisingly), caregivers increased their levels of irritability, tiredness and stress, and this was associated with negative changes in children's emotions and behaviors.

In a related study that has implications for social isolation experienced by children during COVID-19, Gil-Mardrona et al. examined the impact of the presence or absence of siblings as well as participation in extracurricular activities on motor development. The authors used the checklist of Psychomotor Activities (CPA) to assess multiple aspects of motor behavior such as postural control, balance, and coordination in almost 700 Spanish children aged 5 years old. They determined that participation in extracurricular activities—experiences that essentially disappeared during the pandemic—provided a benefit to children regardless of their sibling status. When taken together with the Huang et al. study, this suggests interactions with other children are a key component of motor development and, more importantly, the *lack* of these interactions (as experienced during the pandemic) has a detrimental effect on the developing motor system.

For many investigators, mandates restricting interpersonal interactions and closing schools resulted in a pause of their in-person research. At the same time, several authors seized on the opportunity that this provided by pivoting from in-person to on-line experimental designs. For example, Weiss et al. examined the impact of modifying program delivery from in-person to hybrid/on-line in the well-established physical activity and life skills program for girls aged 8–14 years called “Girls on the Run” (GOTR), which is an example of the type of extracurricular physical activity programming that has a positive impact on motor development as described by Gil-Mardrona et al. They surveyed over 2,000 caregivers and coaches about the impact of modified lessons, training and program delivery on the experiences and impact of the GOTR program. Caregivers and coaches reported that GOTR had a positive impact on the participants health and wellbeing in a variety of ways, despite changes in formatting due to the pandemic. Further, this research showed that the GOTR program can be successful in a variety of modalities and conditions.

Scott-Andrews et al. also used an online methodology because of the pandemic. In this study, the authors converted an in-person study to an online one. Approximately 200 families participated, and measurements of motor skills were taken using videos on motor skills, physical activity using accelerometers mailed to families, online skill perception questionnaires, and zoom interviews on beliefs about physical activity. The authors conclude that online research is possible, but several challenges needed to be addressed, including recruitment, data collection process, and data quality, among others. They concluded that researchers must develop technologies to facilitate these processes in the future.

In another study that took advantage of on-line learning, Xia et al. examined a large sample (over 45,000) of college aged participants fitness level after participating web-based physical education. Their web-based physical education program was planned to support student's health and wellbeing during the COVID-19 pandemic. This research, implemented in China, showed that during pandemic the percentage of students with “normal” weight decreased, and concomitantly, students' overall Body Mass Index rose. Further, male students' running performance decreased in 50 meters dash as well as 1 000 m run, as did female students' 800 meter running performance. Using an exceptionally large number of participants, this important study showed that physical fitness levels in college aged students decreased without appropriate support given by physical education professionals. This suggests that there is need to create feasible online physical education lessons for any future emergent threats such as a pandemic.

During the pandemic, it was recommended that people spend as much time as possible outdoors. Kjønniksen et al. investigated the effect of two outdoor school ground environments: a constructed courtyard, which offered 44 m^2^ space per child, and a natural forest, which offered 50, 6 m^2^ per child, on the amount of time spent in moderate to-vigorous physical activity (MVPA) of Norwegian fifth- and seventh-grade children. The two environments offered different spaces and opportunities for movement to be physically active outdoors during the school day. The results showed that on average, the children engaged in MVPA for 50 percent of the 60-min period when playing in the two environments. Further, the two different environments contributed equally to the daily amount of MVPA of the children. The findings have the potential to inform policies and programs about the value of outdoor environments in promoting recommended levels of PA among school children when indoor environments are unavailable or present health risks, such as during the pandemic.

Children's basic motor skills have been described as a milestone of their physical activity and are fundamental to healthy development, and it is imperative to have a variety of ecologically valid assessment tools that have been validated in multiple countries. Chang et al. conducted a validation study of the Canadian Agility and Movement Skill Assessment (CAMSA) test using Rash analysis with Chinese children in Zunyi Province. This assessment model is close to “real sports situations” because the required movements are consistent, continuous and extremely similar to movement practice and is used extensively by the Canadian Assessment of Physical Literacy (CAPL). With a large number of participants (1,094 children between the ages of 9 and 12 years old), the results showed significant differences in adequacy and between genders in differential item functioning and difficulty with personal ability.

Motor performance (MP) of children is also important for active lifestyle and health and proper monitoring is important to implement specific programs. Eberhardt et al. propose a best-practice tool that can be used directly by trained teachers (fitness barometer). This tool was studied from 2012 to 2020 with children aged 6–18 years. Results showed that 12.7 percent of children were overweight or obese, and differences emerged between age groups. In 2020, the mean values of endurance and speed [*F*_(4,19,23)_ = 224.81] decreased, again demonstrating the negative impact that the COVID-19 pandemic had on physical activity levels in children.

When looking across the studies in this section, several important points are clear. First, the COVID-19 pandemic caused a major disruption in the ways everyone interacted with others both in and outside of schools. Opportunities for movement experiences in the form of physical education, sporting activities, and unstructured play were all drastically reduced for 12–24 months or more, resulting in dramatic shift in our approaches to physical activity. To that point, the body of research showed that the impact of this shift affected different individuals differently, with some groups (e.g., first born children) suffering larger delays than others. Finally, this body of research revealed some of the unique ways in which diverse groups, cultures, and countries pivoted practices to accommodate COVID restrictions and keep individuals active. The variety of experimental designs and research practices provides a strong statement for human resiliency and ingenuity promoting motor development in the midst of world-wide pandemic.

## Author contributions

All authors listed have made a substantial, direct, and intellectual contribution to the work and approved it for publication.

## Conflict of interest

The authors declare that the research was conducted in the absence of any commercial or financial relationships that could be construed as a potential conflict of interest.

## Publisher's note

All claims expressed in this article are solely those of the authors and do not necessarily represent those of their affiliated organizations, or those of the publisher, the editors and the reviewers. Any product that may be evaluated in this article, or claim that may be made by its manufacturer, is not guaranteed or endorsed by the publisher.

